# Rapid visual detection of anisakid nematodes using recombinase polymerase amplification and SYBR Green I

**DOI:** 10.3389/fmicb.2022.1026129

**Published:** 2022-12-02

**Authors:** Xiuqin Chen, Lianjing Zhao, Jiahui Wang, Haolu Wang, Yangyuan Qiu, Zijian Dong, Chunling Zhang, Mingyuan Liu, Xuelin Wang, Xue Bai

**Affiliations:** ^1^State Key Laboratory of Zoonotic Diseases, Key Laboratory for Zoonosis Research of the Ministry of Education, Institute of Zoonosis, and College of Veterinary Medicine, Jilin University, Changchun, China; ^2^Institute of Animal Husbandry and Veterinary Medicine, Fujian Academy of Agricultural Science, Fuzhou, China; ^3^China National Center for Food Safety Risk Assessment, Beijing, China; ^4^Jiangsu Co-innovation Center for Prevention and Control of Important Animal Infectious Diseases and Zoonoses, Yangzhou, China

**Keywords:** Anisakids, recombinase polymerase amplification, SYBR Green I, visualization, detection

## Abstract

Anisakidosis is a food-borne parasitic disease (FBPD) caused by the third-stage larvae of the family Anisakidae. Therefore, it is important to develop a simple, rapid and equipment-free detection method for anisakids in fish samples or seafood since current methods are time-consuming and require complex instruments. In this study, a recombinase polymerase amplification (RPA)-based method was established for the first time to detect anisakids by targeting the internal transcribed spacer (ITS) regions. The detection results were visualized by including SYBR Green I (SG) in the method. The sensitivity of RPA-SG assay was 10^2^ copies per reaction of recombinant plasmid (within 20 min at 37°C), similar to quantitative real-time PCR (qPCR). The assay had high specificity for detecting anisakids against other related parasites and host fish. In addition, the assay was further used to detect fresh marine fish contaminated with anisakids and it showed high precision. These results indicate that the novel RPA-SG assay suitable for visual detection of anisakids in the field and food safety control.

## Introduction

Food-borne parasitic diseases (FBPDs) have caused a growing public health concern worldwide. FBPDs are increasing due to the increased habit of eating raw or undercooked food. Anisakidosis is one of the common emerging FBPDs. It is caused by the accidental ingestion of raw or inadequately cooked marine fish or squid contaminated by the third-stage (L3) larvae of the family Anisakidae (Hochberg and Hamer, 2010; Shamsi and Suthar, 2016). Most human anisakidosis infections are caused by members of the genera *Anisakis* and *Pseudoterranova* (Mattiucci et al., 2013; Qin et al., 2013; Carrascosa et al., 2015; Adroher-Auroux and Benítez-Rodríguez, 2020). Anisakidosis is mainly characterized by gastric, intestinal, ectopic, and allergic responses (Nieuwenhuizen, 2016; Roca-Geronès et al., 2020). Meanwhile, anisakids can also cause tumors (Corcuera et al., 2018). About 90% of anisakidosis cases occur in Japan, and the remaining occur in Korea, China, France, Spain, and Italy (Mattiucci and D’Amelio, 2014). A joint FAO/WHO expert meeting showed that anisakis is one of the top five parasites that significant affect the trade (FAO/WHO, 2014). Therefore, marine fish or squid should be cooked at above 60°C for more than 1 min or frozen whole at −20°C for more than 24 h to kill the larvae and prevent anisakiasis (EU, 2011; Su et al., 2013). However, dead *Anisakis* larvae may still be allergenic to humans (Golden et al., 2022). Therefore, a rapid, simple and instrument-free method is needed for the timely detection of anisakids in fish or fish-derived food.

Visual examination methods (candling, pressing, and transillumination with UV) are the current methods used for anisakid detection (Gómez-Morales et al., 2018). However, these methods have low efficiency and cannot be used to analyze processed fish products (surimi, canned and salted products) (Cammilleri et al., 2020). As a result, molecular techniques have been used as an alternative. For example, various polymerase chain reaction (PCR)-based methods have been widely applied to detect anisakids, and showed satisfactory sensitivity and specificity (Fang et al., 2010; Lopez and Pardo, 2010; Godínez-González et al., 2017; Paoletti et al., 2018). However, these techniques mainly depend on expensive laboratory equipment and personnel training, thus limiting their applications. To circumvent the limitations of PCR-based methods, isothermal amplification techniques, including loop-mediated isothermal amplification (LAMP), rolling circle amplification (RCA), nucleic acid sequence-based amplification (NASBA), strand displacement amplification (SDA), helicase-dependent amplification (HDA), and recombinase polymerase amplification (RPA) (Lobato and O'Sullivan, 2018; Kumar, 2021) were proposed. Unlike PCR-based methods, isothermal amplification methods can be performed at a constant temperature. Moreover, these isothermal amplification methods can be combined with various microdevices, such as paper lateral flow test strips or colorimetric and fluorescence assays, making them suitable for point-of-care or on-site detection (Kumar et al., 2017).

RPA is a robust, sensitive, and specific isothermal technique that achieves exponential amplification using recombinant enzymes, single-stranded binding protein (SSB), and strand-displacing DNA polymerase (Piepenburg et al., 2006). Furthermore, RPA does not need expensive instruments since the reactions can be triggered by human body heat or fist (Crannell et al., 2014; Wang et al., 2017). RPA can be applied in low-resource settings, and used as a point-of-care test due to the low operation temperature (25–42°C), and it is time effective (results in 5–20 min) (Daher et al., 2016). Although the method has been used in some fields (detection of pathogens (Zhang et al., 2020; Wang W. et al., 2021; Wang X. et al., 2021; Wang Z. et al., 2021; Azinheiro et al., 2022), drug resistance gene (Nakano et al., 2018), and genetically modified crops (Wang et al., 2017, 2020), it has not been used for anisakid detection.

In this study, an RPA method combined with SYBR Green I (SG) was proposed for the rapid detection of anisakids by targeting the internal transcribed spacer (ITS) regions of nuclear ribosomal DNA (rDNA). The assay had high sensitivity and specificity for the detection of anisakid in contaminated fish samples. Meanwhile, the method does not require costly PCR instruments, thus significantly shortening the sample-to-answer turnaround time. To the best of our knowledge, this is the first study to use RPA-SG assay to visually detecting anisakids in fish samples using a mini-UV flashlight.

## Materials and methods

### Parasite collection and DNA extraction

The L3 stage larval worms of anisakids were isolated from the viscera of *Trichirurus lepturus*. Fish samples were sourced from a fish market in the coast of Fuzhou, China. The isolated larvae were washed using physiological saline (pH = 7.4). A light microscopy (Nikon SMZ 800) was used to observe the larvae for the identification at genus level. Then larvae were fixed in 70% ethanol at-20°C for species identification using PCR-RFLP of the ITS (D’Amelio et al., 2000). TIANamp Genomic DNA Kit (Tiangen Biotech, Beijing, China) was used to extract genomic DNA from each worm following the manufacturer’s protocol. Spectrophotometry was used to determine the purity and concentration of DNA *via* a Nanodrop (Thermo Scientific, Wilmington, DE).

### Primer design

The conserved regions of the ITS were identified by aligning nucleotide sequences of *Anisakis* spp. using the MEGA 11.0. The duplex between primer pairs was analyzed using Primer Premier 5.0. A pair of RPA primers of anisakids species was designed following the TwistAmp Assay Design Manual. A Primer Express software (v 2.0; Applied Biosystems) was used to design a pair of TaqMan-based quantitative real-time PCR (TaqMan qPCR) primers and a probe targeting the ITS regions. The primer and probe sequences are shown in Table 1. The primers and probe were synthesized at Sangon Biotech Biotechnology Co., Ltd., (Shanghai, China).

**Table 1:** Primers and probe used in RPA assay and qPCR for anisakid detection.

Target gene	Name	Sequence	Amplicon size
ITS	RPA-F	5’-AATTGCTGTTGTGTTGTTGGTGATTCTATCA-3’	192 bp
	RPA-R	5’-ATCACGTATGCTGGTTGTTGCCCCTATGAA-3’	
	qPCR-F	5’-AGCGAATCCAAAACGAACGA-3’	80 bp
	qPCR-R	5′- GAGTTTCCATGTGGCTCACAAC-3’	
	qPCR-P	VIC**-**5’-TCTCCCAACGTGCATAC-3’-MGB	

ITS, internal transcribed spacer; MGB, minor groove binding

### Preparation of the recombinant plasmid

PCR was used to amplify the rDNA ITS sequence of anisakids. The PCR products were cloned into pUC57 vector to construct a standard plasmid using DNA Ligation Kit and *E. coli* Competent Cell JM109 (TaKaRa Biotechnology, Dalian, China). An E.Z.N.A. Plasmid Mini kit (Qiagen, Hilden, Germany) was used to purify the samples. A spectrophotometer was used to determine the concentration and purity of plasmid. The number of recombinant plasmid copies was calculated as follows: (copies/μl) = 6.02 × 10^23^ × 10^−9^ × concentration/ (fragment length × 660). Ten-fold serial dilutions of the recombinant plasmids (1.29 × 10^8^ − 1.29 × 10^1^ copies/μL) were prepared, and aliquots of each dilution were frozen at-20°C.

### RPA assay for anisakids detection

RPA assay was conducted using a TwistAmp® Basic kit (TwistDx, Cambridge, UK). Each reaction contained 29.5 μl of rehydration buffer, optimal concentration of primers, 2.5 μl magnesium acetate solution, and 1 μl template. Nuclease-free water was added to make the total volume (50 μl). Negative control was set for each RPA assay. Three primer concentration gradients (0.2, 0.36 and 0.72 μM), three reaction time gradients (10, 15 and 20 min) and seven temperature gradients (25, 30, 33, 35, 37, 39, 42°C) were tested to optimize RPA reaction conditions following the manufacturer’s instructions.

Amplicons (25 μl) were purified using a TIANquick Midi Purification kit (Tiangen Biochemical Technology, Beijing, China) after amplification. The purified products (9 μl) were electrophoresed on 2% (w/v) agarose gel at a constant voltage (5 V/cm) for 30 min. The products were then visualized under UV light.

### TaqMan qPCR detection

A Mastercycler ep realplex system (Eppendorf, Germany) was used for TaqMan qPCR assay. Each reaction contained 10 μl Premix Ex Taq Probe qPCR (TaKaRa Biotechnology, Dalian, China), 0.5 μl of each primer (10 μM), 0.5 μl of probe (10 μM), and 1 μl of template. Nuclease-free water was added to make the total volume 20 μl. Thermocycler settings were as follows: 95°C for 2 min; 40 cycles at 95°C for 15 s, and 60°C for 45 s. All reactions were performed in triplicates. A standard curve was generated from serially diluted recombinant plasmids. The results were positive if Ct < 35 with a sigmoid-shaped amplification curve and negative if Ct > 35.

### Sensitivity and specificity analysis

For sensitivity analysis, the recombinant plasmid was 10-fold serially diluted to achieve DNA concentrations of 1.29 × 10^8^ − 1.29 × 10^1^ copies/μl. Each recombinant plasmid dilution (1 μl) was used as a template and amplified using the RPA or TaqMan qPCR assay.

Specificity was assessed using some related fish parasites, including *Hysterothylacium aduncum* and *Ligula intestinalis*. Furthermore, verified larvae-free flesh of *Trichirurus lepturus* and *Larimichthys polyactis*, which are hosts of the L3 stage of anisakids, were used to avoid the amplification of fish host DNA.

### Naked-eye visualization

Upon completion, 2 μl of 400 × SG (10,000 × stock solution, Solarbio Science & Technology Co., Ltd., Beijing, China) was added to the remaining 25 μl of RPA products. The amplicons of positive samples emitted green fluorescence while the negative samples remained colorless.

### Verification of the feasibility

Five farmed *Larimichthys crocea* samples were sourced from a local supermarket. qPCR was used to detect the absence of anisakids. The fish samples were spiked with anisakid as described by Lopez and Pardo (2010) with minor modifications. Briefly, the fish samples were spiked with anisakid larval (0.0005% − 0.05% (w/w)) and homogenized with 25 ml of phosphate-buffered saline (PBS) for 5 min. In parallel, the mixture was subjected to boil for 15 min. The mixture (300 mg) was mixed with 300 μl of extraction buffer [1% (w/v) SDS, 150 mM NaCl, 2 mM EDTA, and Tris–HCl at pH 8.0] supplemented with 10 μl of 5 M guanidine thiocianate and 10 μl of proteinase K, then incubated at 56°C for 1.5 h. Genomic DNA of the supernatant was extracted using a TIANamp Genomic DNA Kit (Tiangen Biotech, Beijing, China) to determine the relative LOD of the RPA-SG assay. The non-spiked genomic DNA extracted from fish samples was used as a negative control. TaqMan qPCR assay was performed in parallel. DNA was extracted and detected from each treatment in triplicates.

## Results

### Optimization of RPA assays

**Figure 1:**
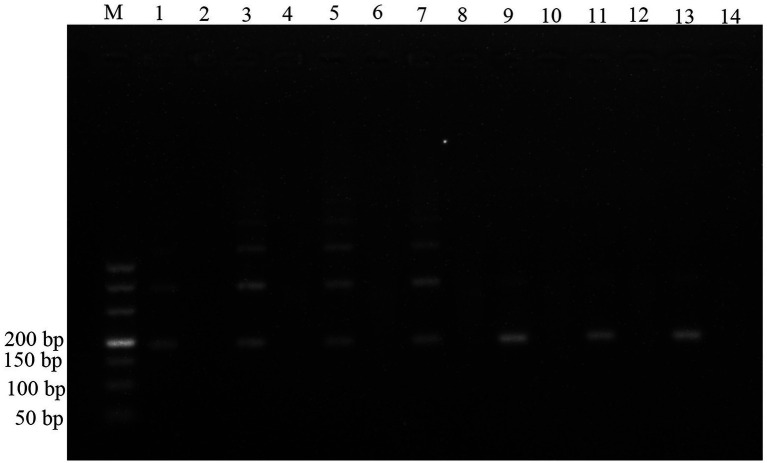
Optimization of reaction temperature for the RPA assay. Lane M: DL500; lane 1: 25°C; lane 3: 30°C; lane 5: 33°C; lane 7: 35°C; lane 9: 37°C; lane 11: 39°C; lane 13: 42°C; and lanes 2, 4, 6, 8, 10, 12, 14 were the negative controls (NTC) corresponding to the reaction temperature.

RPA reaction temperatures, times, and primer concentrations were evaluated to achieve the best RPA performance. Seven groups of RPA reactions testing 1.29 × 10^7^ copies/μl standard plasmid were individually incubated at 25, 30, 33, 35, 37, 39, and 42°C. Non-target bands were yielded at 25, 30, 33, and 35°C, failing to meet the experimental requirements (Figure 1). In contrast, expected size amplicons were visualized in agarose gels after incubation at 37, 39, and 42°C. The assay is easy to operate and can be used in resource-limited areas, and thus 37°C was selected as the optimal reaction temperature for anisakid detection.

**Figure 2:**
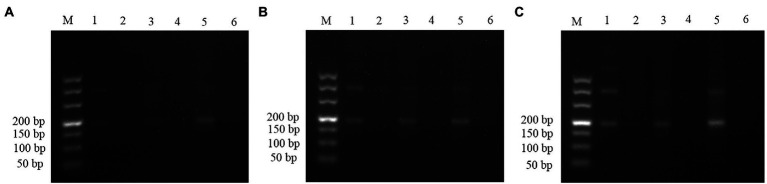
Optimization of incubation time and primer concentrations for the RPA assay. **(A)** Samples incubated for 10 min. **(B)** Samples incubated for 15 min. **(C)** Samples incubated for 20 min. About 1.29 × 10^7^ copies/μL of plasmids were used as template. Lane M: DL500; the odd-numbered lanes represent assays amplified with primers concentrations of 0.2, 0.36, 0.72 μM. The even-numbered lanes represent the negative controls (NTC) of each primer concentration.

The plasmids (1.29 × 10^7^ copies/μl) were used as the templates to assess the amplification efficiency at the optimal reaction temperatures, three incubation times (10, 15 and 20 min), and three primer concentrations (0.2, 0.36, and 0.72 μM). The RPA assay could not yield any visible target fragment at 10 min (Figure 2A). However, the target fragment became brighter with the extension of incubation time (Figures 2B,C); thus, 20 min was selected as the optimal incubation time. The three primer concentrations amplified the target band (Figure 2). However, the primer concentration of 0.72 μM was superior to 0.2 and 0.36 μM, thus being the most suitable.

### Sensitivity of the RPA assay

**Figure 3:**
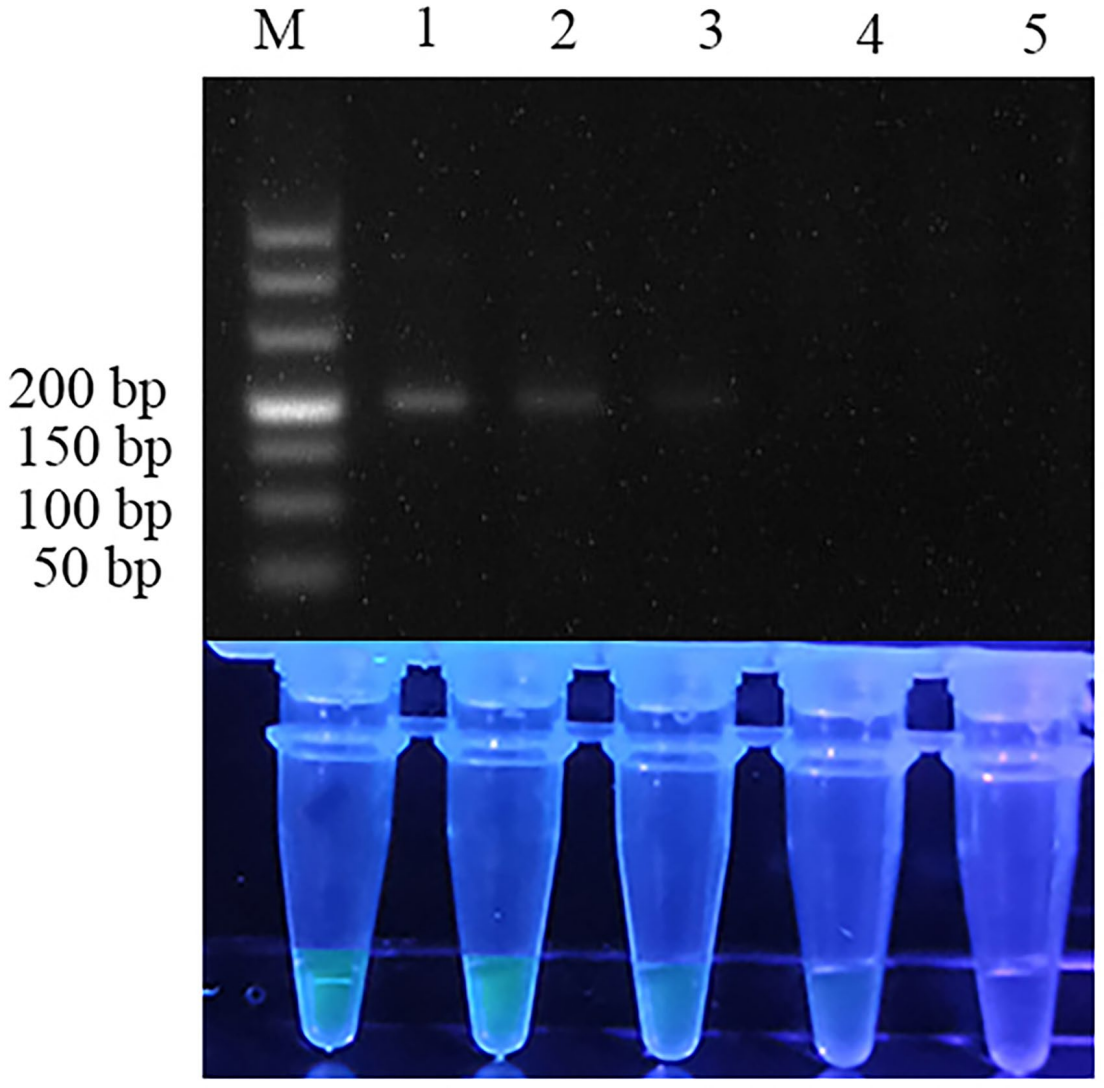
Sensitivity of RPA assay. From left to right, the initial templates were 10-fold serially diluted from 1.29 × 10^5^ to 1.29 × 10^2^ copies per reaction, with no template added as a negative control. Lane M: DL500. Top row: agarose gel electrophoresis images of RPA reactions. Bottom row: Fluorescent assay images corresponding to the RPA reactions. The sensitivity of the gel electrophoresis images and visual detection images of RPA were 10^3^ and 10^2^ copies per reaction, respectively.

**Figure 4:**
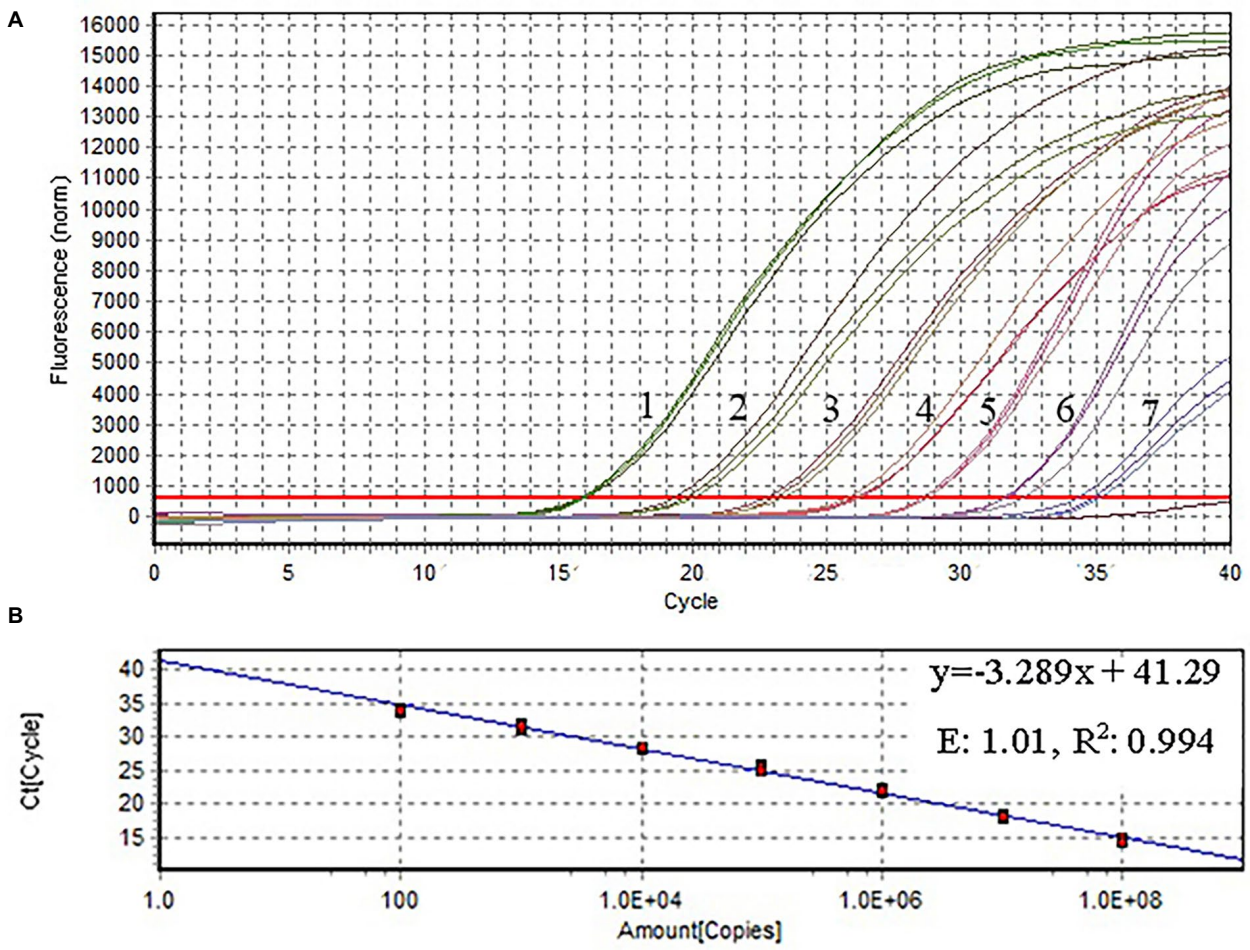
Amplification plot and standard curve of qPCR. The *X*-and *Y*-axes represent copies of the plasmids, and cycle threshold (Ct), respectively. *R*^2^: coefficient of determination; E: efficiency. The assay was performed using serial 10-fold dilutions of the plasmid DNA standard (1.29 × 10^8^–1.29 × 10^2^ copies/μl). Plasmid concentration and threshold cycles had a linear relationship. The lowest copy number that could be determined by qPCR was 1.29 × 10^2^ copies/μl.

The pUC57 plasmid carrying the ITS gene was used as a template to assess the sensitivity of the assay. Agarose gel electrophoresis images of RPA assay could detect 1.29 × 10^3^ copies of recombinant plasmid per reaction (Figure 3). SG was used to analyze the RPA products *via* a mini-UV torch. The positive samples were bright green under UV light (395 nm), while the negative samples remained colorless (Figure 3). The visual detection of RPA images achieved a limit of detection (LOD) of 10^2^ copies per reaction, which was 10 times more sensitive than that of gel electrophoresis images of RPA assay. TaqMan qPCR assay was performed for comparison purposes. The qPCR assay could detect 1.29 × 10^2^ copies per reaction, similar to RPA-SG assay (Figure 4).

### Specificity of the RPA assay

**Figure 5:**
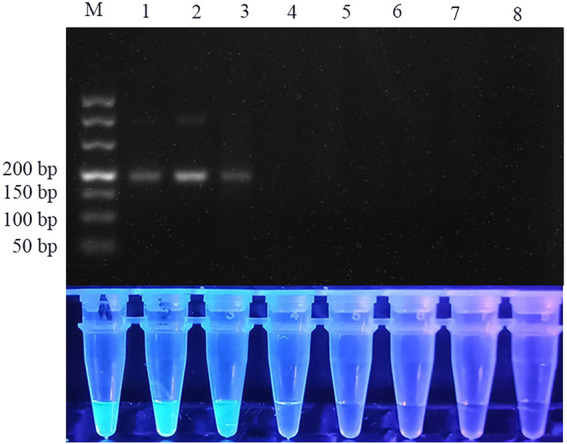
Specificity of RPA assay. Lane 1: *Anisakis simplex*; lane 2: *Anisakis pegreffi*i; lane 3: *Anisakis typica*; lane 4: *Hysterothylacium aduncum*; lane 5: *Ligula intestinalis*; lane 6: *Trichirurus lepturus* tissue; lane 7: *Larimichthys polyactis* tissue; lane 8: ddH_2_O. Top row: agarose gel electrophoresis images of RPA reactions. Bottom row: Fluorescent assay images corresponding to the RPA reactions.

Other related fish parasites and host fish were used to further evaluate the specificity of the two assays. RPA assay detected the target bands at the right position (192 bp) in three ansakids (*Anisakis simplex*, *Anisakis pegreffi*i, *Anisakis typica*), while *Hysterothylacium aduncum*, *Ligula intestinalis*, *Trichirurus lepturus* tissue, *Larimichthys polyactis* tissue and no template control were negative (Figure 5). As expected, the specificity of the RPA-SG assay was consistent with the qPCR assay, demonstrating the RPA-SG could effectively distinguish anisakid from other parasites high specificity (Supplementary Figure S1).

### Detection of anisakis in artificially contaminated fish samples *via* RPA-SG

*Larimichthys crocea* samples were spiked with different proportions of anisakid larval (0.0005% − 0.05% (w/w)) to assess the feasibility of the RPA-SG assay. The fish samples were boiled for 15 min in parallel since the assay may be applied to processed food. The samples were simultaneously tested using the qPCR assay to verify the RPA-SG results. RPA-SG results were consistent with the qPCR results, except for the samples spiked with 0.001% of anisakid larval without heating and the 0.005% of boiled samples (Table 2). Fish samples spiked with 0.005% of anisakid larval had positive fluorescence signals. However, no fluorescence signals were observed when the spike proportion was less than 0.001%. Therefore, the minimum contamination proportion that could be detected by RPA-SG assay was 0.005% of anisakid larval in fish samples, indicating high sensitivity. However, the detection sensitivity decreased to 0.01% for the boiled fish samples. These results indicate that the sensitivities of RPA-SG assay and qPCR assay are almost similar.

**Table 2:** Anisakid detection in artificially contaminated fish samples analyzed by RPA-SG and qPCR assay.

Concentration^*^(%)	Without heating		Boiling for 15 min	
	RPA-SG	qPCR (Cq)^#^	RPA-SG	qPCR (Cq)^#^
0.0005	−	35.46 ± 0.22	−	36.73 ± 0.24
0.001	−	34.70 ± 0.19	−	35.90 ± 0.18
0.005	+	32.37 ± 0.17	−	33.55 ± 0.20
0.01	+	31.55 ± 0.15	+	32.14 ± 0.18
0.05	+	29.53 ± 0.18	+	30.73 ± 0.20

“+” = positive results, “–” = negative results. ^*^Marine fish samples were spiked with different concentration of anisakid larvae (w/w). ^#^ The data are presented as mean ± standard deviation (SD). Each concentration had three triplicates.

## Discussion

Human anisakidosis is an emerging FBPD worldwide, especially in countries with high marine fish consumption due to the increased intake of raw or undercooked fish-derived food (Sashimi and sushi). This could also be due to the increasing prevalence of larvae of anisakid nematodes in some fish species (Herrero et al. , 2011). The European Food Safety Authority has shown that the larvae generally can be found in fishery products, with a “presumption of infection” (Herrero et al., 2011). Therefore, a reliable, simple and time-effective should be developed to prevent the dissemination of anisakids in the food chain.

The genetic marker rDNA ITS regions (Fang et al., 2011; Cavallero et al., 2017; Cammilleri et al., 2020) or cytochrome c oxidase II (COX II) gene (Lopez and Pardo, 2010; Onzález et al., 2021) is widely used to detect anisakids larvae. Compared with COX II, rDNA ITS is more variable among anisakid species. In this study, RPA-SG method was established using rDNA ITS regions as a target for anisakids detection. RPA reaction has a high background signal in the negative samples when the amplification time and concentration of primers are increased (Piepenburg et al., 2006). Herein, RPA reaction conditions including temperature, duration, and primer concentration were optimized to reduce the background signal. Distinct fluorescent results were obtained using naked eye with the help of a mini-UV torch.

The sensitivity of RPA-SG assay was 10^2^ copies per reaction, similar to that of the qPCR assay. However, once the RPA-SG assay was applied to spiked samples detection, the sensitivity decreased slightly. This is probably result from the background DNA in fish samples. Previous studies reported that RPA is able to amplify target nucleic acids in the presence of background DNA. However, the tolerability is concentration dependent (Chao et al., 2015; Clancy et al., 2015; Rohrman and Richards-Kortum, 2015). Clancy et al. found that the RPA reaction was inhibited by the presence of background DNA, varying from no significant inhibition to substantial inhibition. However, the PCR was not inhibited with the same amount of background DNA (Clancy et al., 2015). Because the principle of nucleic acid amplification of qPCR is similar to that of PCR. Therefore, the qPCR assay may be less inhibited by background DNA than RPA-SG assay. Although the sensitivity of the RPA-SG assay was lower than that of qPCR assay in clinical samples detection, the assay was time-saving, friendly, and instrument-free. Additionally, the thermal treatment decreased the sensitivity of the assay, similar to previous studies (Herrero et al., 2011; Prieto et al., 2014).

The developed RPA-SG assay could successfully detect anisakids in 20 min at 37°C. Unlike other molecular detection methods (PCR and qPCR), the RPA-SG assay requires a significantly shorter time (Fang et al., 2011; Herrero et al., 2011). In previous studies, it took 2.5–4 h to complete the entire detection of ansakids (Fang et al., 2011; Herrero et al., 2011). In addition, the RPA-SG assay and qPCR assay had similar sensitivity, specificity and flexibility for the detection of fish samples. Furthermore, Cammilleri et al. (2020) developed a LAMP assay to detect *Anisakis* spp. for 35 min at 65°C. However, the reaction required a heater. Moreover, primer designing is complex. However, the reaction in the RPA-SG assay does not require a heater and is thus suitable for on-site detection and point-of-care test (Crannell et al., 2014). Although RPA-SG assay is more costly than PCR-based methods, it saves time.

However, RPA assay associated with aerosol contamination due to production of amplicons. Therefore, the addition of templates and transfer of amplification products should be conducted in different rooms (Lu et al., 2022). Designing portable and contamination-prevention cartridges can also prevent contamination. Therefore, on-site detection of anisakids with a convenient and compact cartridge that completely contains the reaction is crucial for characterizing the assay performance.

In summary, a rapid and visible RPA-based method was developed to detect anisakids species. The assay had good sensitivity and was easy to operate. Therefore, the assay could be an alternative to PCR-based methods for rapid and on-site detection of anisakids. The assay can prevent anisakiasis, thus ensuring food security control.

## Data availability statement

The original contributions presented in the study are included in the article/Supplementary material, further inquiries can be directed to the corresponding authors.

## Author contributions

XC, LZ, and JW performed experiments. HW, YQ, and ZD participated in collecting the samples. CZ and ML analyzed the data. XC drafted the manuscript. XB and XW conceived and designed the study, and critically revised the manuscript. All authors contributed to the article and approved the submitted version.

## Funding

This study was supported by the National Key Research and Development Program of China (2021YFC2600200), National Natural Science Foundation of China (62101209), Interdisciplinary Integration and Innovation Project of JLU (JLUXKJC2021ZZ10) and China Postdoctoral Science Foundation (2022 M710057). The funders had no role in study design, data collection and analysis, decision to publish, or preparation of the manuscript.

## Conflict of interest

The authors declare that the research was conducted in the absence of any commercial or financial relationships that could be construed as a potential conflict of interest.

## Publisher’s note

All claims expressed in this article are solely those of the authors and do not necessarily represent those of their affiliated organizations, or those of the publisher, the editors and the reviewers. Any product that may be evaluated in this article, or claim that may be made by its manufacturer, is not guaranteed or endorsed by the publisher.
