# Supplementary figures and images for: Rapid visual detection of anisakid nematodes using recombinase polymerase amplification and SYBR Green I
Source: Front Microbiol. 2022 Dec 2;13:1026129. doi: 10.3389/fmicb.2022.1026129 (PMC9756439; doi:10.3389/fmicb.2022.1026129)

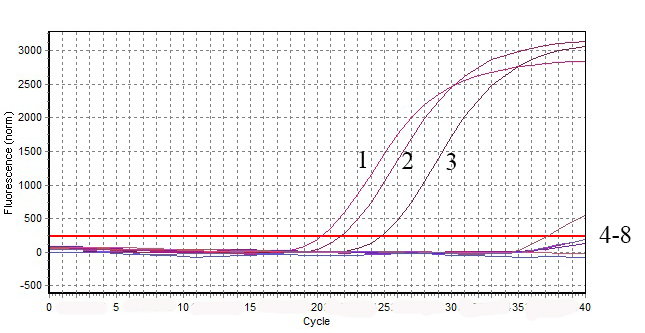

Supplement: Supplementary file 1 [file Image_1.JPEG]
